# Physicochemical characterization of vibriophage N5

**DOI:** 10.1186/1743-422X-2-27

**Published:** 2005-04-11

**Authors:** Anindito Sen, Amar N Ghosh

**Affiliations:** 1 Division of Electron Microscopy, National Institute of Cholera and Enteric Diseaess, P-33, C.I.T. Road, Scheme- XM, Beleghata, Kolkata- 700010. India; 2 (Present Address)Laboratory of Structural Biology, Room 1504, Building 50, NIAMS/NIH Bethesda, MD, 20852, USA

**Keywords:** Vibriophage N5, DNA, *Vibrio cholerae*, Electron Microscopy, Partial denaturation mapping, Bacteriophage

## Abstract

Phage N5 is one of the phages of *Vibrio cholerae *serovar O1 biotype El Tor (Ghosh, A. N., Ansari, M. Q., and Dutta, G. C. Isolation and morphological characterization of El Tor cholera phages. *J. Gen. Virol*. 70: 2241–2243, 1989). In the present communication the growth curve, molecular weight and confirmation of the genome, partial denaturation map and restriction endonuclease digestion pattern have been determined. Partial denaturation map indicates that the genome has non-permuted / invariant sequence. Presence of cohesive ends has also been documented.

*Vibrio cholerae*, the causative agent of cholera in humansis classified into two serotypes: O1 and nonO1 [1]. The O1 strains are divided into two biotypes: Classical and El Tor. Before 1961 most epidemics had been caused by the classical biotype. However after 1961 the El Tor strains became the main causative agent of the cholera. Phage typing has proved to be useful and successful tool to tract down the spread of this dreadful disease. Vibriophage has also proved to be useful in studying the host chromosomes [2]. In the present work we report physicochemical characterization of an ElTor vibriophage N5 that was isolated from the sewage water samples of Calcutta, India [3].

The N5 phage was isolated from the sewage water of Calcutta [3] and was propagated on MAK 757, a *Vibrio cholerae *O1 ElTor strain. One-step growth curve of this phage was determined following the method described in Adams [4]. About 10^5 ^cells of freshly cultured MAK 757 were infected with N5 phage at an m.o.i. of 0.1. An aliquot was withdrawn at every 5 minutes and titrated for the total number of phages. A total of 8 × 10^8 ^plaque forming units are generated after 50–55 minutes from the time of infection. The eclipse period is nearly 8–10 minutes.

A phage lysate of N5 was prepared on soft agar (1% Nutrient Agar, pH 7.4, 0.5% NaCl, 1.5% Agar, HiMedia laboratories, Mumbai, India) overlay using freshly cultured MAK 757 (m.o.i of 0.01) as the propagating strain [4]. A few drops of chloroform were added to the freshly prepared phage lysate to remove bacterial content in it. The phage lysate (nearly 10^9^phages/ml) was subjected to ultracentrifugation at 35,000 r.p.m. for 1 h and 30 mins in a Sorval T 865 rotor and a phage pellet was obtained. The phage pellet was resuspended in 1 ml of 50 mM – Tris-HCl pH 7.5, 20 mM – MgCl_2 _(TM buffer) to concentrate and the phage was stored at 4°C. The phage was purified on a sucrose step gradient of 10% to 40% as described previously [5,6] using a Sorval TW 668 swing-out rotor at revolution speed of 35,000 r.p.m. for 1 h and 15 minutes. The purified phage pellet l was re-suspended in 1 ml of TM buffer and stored at 4°C. The final concentration the resuspension was nearly 10^11 ^phages/ml.

The N5 phage has an isomeric head with an extremely short non-contractile tail (figure 1a inset). The diameter (distances between the opposite apices) of the of the head is nearly equal to 71.5 ± 1.5 nm and the length of the tail is equal to 12.2 ± 1.9 nm. The tails are so short that in many of the phages, when observed under electron microscope, the tails are not visible because of the improper orientations on the specimen support film or breakage during phage preparation. N5 phage belongs to the 'podoviridae' family according to international committee for the taxonomy of viruses (1982).

**Figure 1:**
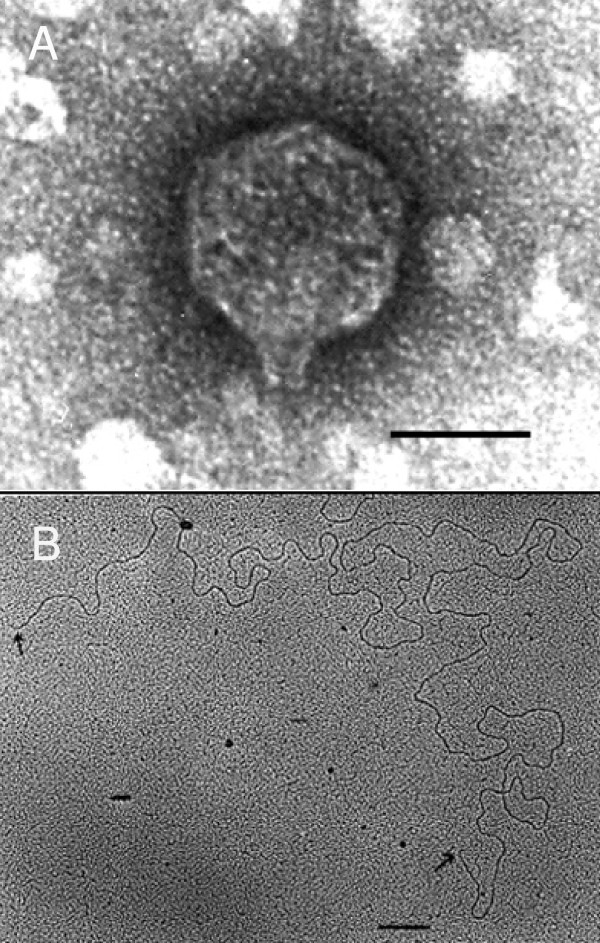
Electron microscopic analysis of vibriophage N5 virion morphology and DNA structure. Panel A: Electron micrograph of vibriophage N5 stained with 2% uranyl acetate. Bar: 40 nm. Panel B: Electron micrograph of N5 DNA mounted on the grid by Kleinschmidt's technique. The arrows show the free ends of the DNA indicating that it is linear. Bar: 300 nm.

The high-titer purified phage lysate (10^11^/ml) was mixed with equal volume 0.0625 M Tris-HCl (pH 6.8) along with 1% sodium dodecyl sulphate (SDS) 15% glycerol, 1% Beta-mercaptoethanol and bromophenol blue. The solution was then incubated at 100°C for 3 minutes. SDS-polyacrylamide gel electrophoresis (PAGE) was performed by the method of Laemmli [7] as adopted by Sambrook *et al*., [8] for obtaining the SDS-PAGE pattern of the structural proteins of the N5 phage. The apparent molecular masses of the vibriophage N5 polypeptides were evaluated by SDS- PAGE 12.5% step-gel electrophoresis (figure 2a). From figure 2a we find four major bands of sizes 51 kDa, 37 kDa, 25 kDa and 18 kDa respectively. The major component had a molecular size of 51 kDa (approx). However, two small bands of 15 kDa and 12 kDa also visible in the step SDS-PAGE step-gel electrophoresis along with several other extremely faint bands that appeared in figure 2a which are probably bacterial debris.

**Figure 2:**
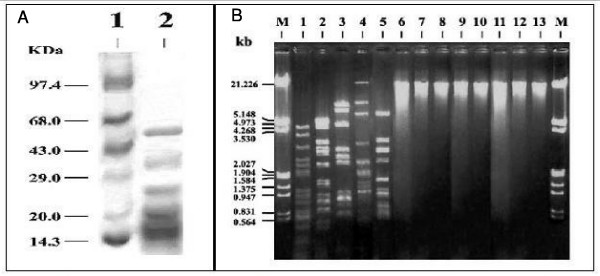
Structural proteins of vibriophage N5 and restriction enzyme analysis of vibriophage N5 DNA. Panel A: SDS-PAGE patterns of structural proteins of purified N5 Vibriophage. Panel B: Restriction endonuclease digestion pattern of the N5 vibriophage DNA.

A comparison of these results with the SDS-PAGE of the N4 phage [6] reveals that the molecular masses of the N5 polypeptides are slightly higher than that of N4 phage. Nevertheless, the second band of 37 kDa in N5 (figure 2a) is very much of the same size as of the 36 kDa of the N4 phage. In fact the SDS-PAGE of the structural proteins of N5 vibriophage closely resembles the SDS-PAGE pattern of another vibrio El Tor phage e5 [9], which also has a 51-kDa polypeptide as a major component.

N5 vibriophage DNA was extracted from the phage using phenol-chloroform method described in Sambrook *et al*., [8] and dialyzed against 20 mM NaCl, 5 mM EDTA, (pH 7.4) buffer. The N5 DNA was spread using protein monolayer technique of Kleinschmidt *et al*. [10] with the modifications described in Inman [11]. About 500 ng/ml of N5 DNA was mixed up with 50 ng/ml of pBR322 marker DNA along with 0.067 M Na_2_CO_3_, 0.0107 M EDTA, 50% formamide (Sigma), and 0.01% cytochrome *c *(Sigma) at pH 7.4. The hypophase was double distilled water. The DNA-bound protein was picked up on carbon coated nickel grids, stained with uranyl acetate and was subjected to rotary shadow with platinum. Figure 1b shows a N5 DNA. The two ends (marked by arrows) are clearly visible thus indicating that the N5 DNA is linear. The length of the N5 phage DNA is computed to be 40.7 ± 0.7 kb as compared with pBR322 DNA of length 4.36 kb used as a marker (not seen in figure 1). This is very much similar to the size of the linear N4 vibriophage DNA that has a size of 40.4 ± 0.1 kb [6].

Partial denaturation of the N5 vibriophage DNA was carried out as described previously [4,8] A high pH buffer was prepared that contained 34% formaldehyde, 10 mM Na_2_CO_3_, 1 mM EDTA and suitable amount of NaOH to make up the pH to 10.9. About 7 μl of N5 DNA was gently mixed with 3 μl of the high pH buffer and was incubated at 37 ± 1°C for 15 minutes. The final solution was then mixed with formamide and cytochrome *c *to a final concentration of 50% and 0.01% respectively. Partial denatured vibriophage N5 DNA molecules were obtained (not shown in figure). The DNA molecules were arranged in a linear fashion according to their denaturation sites and a partial denaturation map was constructed (figure 3a). A weight average, denaturation histogram (figure 3b) of these maps was plotted to visualize the average denaturation pattern of the total N5 DNA molecules. It is quiet apparent from the histogram that there are at least 5 major denaturation sites. There is always a denaturation site at one end (by convention to the right-hand end, Inman, [11]) of the DNA molecule irrespective of the degree of denaturation. The other denaturation sites are at postions 26%, 65 %, 75 % and 91% from the left hand end respectively and a minor peak at the 55% position (figure 3b). The result shows that the vibriophage N5 DNA has a non-permuted and unique sequence. Comparison with the denaturation map of N4 [6] reveals that N5 had denaturation site at one end (right hand end) while N4 DNA has a denaturation sites at both ends. However the vibriophage D10 DNA has denaturation peaks at the same locations as that in N5 phage. In this respect denaturation map of N5 DNA is similar to that of D10 DNA [5].

**Figure 3:**
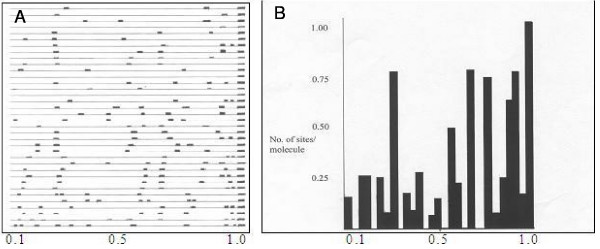
Partial denaturation maps of vibriophage N5 DNA. Panel A: Vibriophage N5 DNA was subjected to partal denaturation. Each line represents one double stranded DNA molecule. The denaturation sites along each DNA molecule are shown by small solid rectangles. Panel B: Histogram average of partial denaturation maps of N5 DNA.

Since the N5 DNA is non-permuted it was expected that the DNA might have cohesive ends [12]. In order to test whether the N5 DNA has cohesive ends 3 μl of N5 DNA (500–800 μg/ml) was mixed with 2 mM Tris, 0.2 mM EDTA buffer (pH 8.4) along with 20 mM Tris, 2 mM EDTA buffer (pH 8.4), 50 mM Na_2_CO_3 _and 50% of formamide [13].

The mixture was left for incubation at room temperature for 72 hrs. After about 48 hrs 2 μl of Tris-EDTA buffer {0.1 M Tris + 10 mM EDTA (pH 8.4)} was added (to maintain the pH of the mixture) to the mixture and left for another 24 hrs of incubation at room temperature. After the completion of 72 hrs of incubation 3 μl of cytochrome *c *was added to a final concentration of 0.01% and was spread on double-distilled water. After examining about 10 DNA molecules it was found that molecules have length nearly twice the native length of the N5 DNA. The average length of these 20 molecules is 79 ± 0.8 kb while is twice the native length mentioned earlier i.e. 40.7 ± 0.7 kb. This confirms that N5 DNA has cohesive ends.

Restriction endonuclease digestion of the N5 vibriophage was carried out with the help of the procedure recommended by manufactures ("Genie", India). The enzymes used were: *Eco *RI, *Sal *I, *Bam *H1, *Bgl *II, *Pst *I, *Bgl *I, *Ass *I, *Sma *I, *Hind *III, *Hpa *II, *Eco *RV, *Acc *I, *Hae *III and *Xba *I. Restriction endonuclease digestion pattern of the N5 phage DNA revealed that the N5 DNA is double stranded. The N5 phage DNA was resistant to *Eco *RI, *Sal *I, *Bam *H1, *Bgl *II, *Pst *I, *Bgl *I, *Ass *I and *Sma *I. It is worth mentioning here that the DNA of vibrio El Tor typing phage 'e5' is also resistant to first five restriction endonucleases mentioned above [9]. However both e5 and N5 phage DNAs have restriction sites of *Hpa *II (figure 2b and [9]). It is also observed that Hind III gives rise to 6 kb, 2 kb, and 1.3 kb common fragments in N5 and N4 [6] but N5 DNA has an additional fragment of 21 kb which is absent in N4 DNA.

## References

[B1] Mukherjee S (1978). Principles and practice of typing *Vibrio cholerae*. Methods Microbiol.

[B2] Guidolin A, Manning PA (1987). Genetics of *Vibrio cholerae *and its bacteriophages. Microbiol Rev.

[B3] Ghosh AN, Ansari MQ, Dutta GC (1989). Isolation and morphological characterization of El Tor cholera phages. J Gen Virol.

[B4] Adams MH (1959). Bacteriophages.

[B5] Chakrabarti BK, Chattopadhyay DJ, Ghosh AN (1993). Vibriophage D10 contains non-permutated DNA with cohesive ends. J Gen Virol.

[B6] Ghosh AN, Chakrabarti BK, Chattopadhyay DJ, Sil S (1995). Vibriophage N4 DNA is nonpermutated and terminally redundant. Can J Microbiol.

[B7] Laemmli UK (1970). Cleavage of structural proteins during the assembly of the head of bacteriophage T4. Nature.

[B8] Sambrook J, Fritsch EF, Maniatis T, Ford N, Nolan C, Ferguson M (1989). Bacteriophage λ growth, purification and DNA extraction.2.60–2.81, and SDS-polyacrylamide gel electrophoresis of proteins, 18.47–18.59. Molecular cloning: a laboratory manual.

[B9] Basu R, Ghosh AN, Ghosh A (1993). Biophysical characterization of Vibrio El Tor typing phage e5. FEMS Microbiol Lett.

[B10] Kleinschmidth AK, Lang D, Jacherts D, Zhan RK (1962). Darstellung und Längenmessungen des gesamten Desoxyriobnuclein-Säure-Inhaltes von T_2 _bakteriophagen. Biochim Biophys Acta.

[B11] Inman RB, Griffith JD (1982). Partial denatuartion mapping of DNA determined by electron microscopy. Electron microscopy in biology.

[B12] Tye BK, Huberman JA, Botstein D (1974). Non-random circular permutation of phage P22 DNA. J Mol Biol.

[B13] Davis R, Simon M, Davidson N (1971). Electron microscope for hetroduplex methods for mapping regions of base sequence homology in nucleic acids. Methods Enzymol.

